# Trait rumination and social anxiety separately influence stress-induced rumination and hemodynamic responses

**DOI:** 10.1038/s41598-022-08579-1

**Published:** 2022-04-01

**Authors:** Hendrik Laicher, Isabell Int-Veen, Florian Torka, Agnes Kroczek, Isabel Bihlmaier, Helena Storchak, Kerstin Velten-Schurian, Thomas Dresler, Ramona Täglich, Andreas J. Fallgatter, Ann-Christine Ehlis, David Rosenbaum

**Affiliations:** 1 Department of Psychiatry and Psychotherapy, Tuebingen Center for Mental Health, University Hospital of Tuebingen, Calwerstraße 14, 72076 Tuebingen, Germany; 2 Department of Psychology, University of Tuebingen, Tuebingen, Germany; 3 LEAD Graduate School & Research Network, University of Tuebingen, Tuebingen, Germany

**Keywords:** Psychology, Human behaviour, Neurophysiology, Cognitive control

## Abstract

We aimed to investigate stress-reactive rumination in response to social stress and its association with social anxiety and trait rumination. From previous investigations we know that people with a certain vulnerability to rumination show increased stress-reactive rumination. However, up to date the possible influence of social anxiety on this relationship is still unclear. Therefore, we reanalyzed the data of two of our previous studies assessing healthy low and high trait ruminators and depressed patients performing the Trier Social Stress Test (TSST). We measured cortical oxygenation using functional Near-Infrared Spectroscopy (fNIRS) as well as different behavioral outcome measures (subjective stress levels, negative affect, state rumination). On a behavioral level, we found an influence of both, social anxiety and trait rumination, on state rumination, even when correcting for the other factor, respectively, implying two potentially independent factors of influence. On a neural level, we observed reduced activation in brain regions of the cognitive control network (CCN) for higher social anxiety and trait rumination, which might be a result of reduced cognitive and attentional control. Results indicate a specific role of social anxiety, at least on a behavioral level, and therefore implicate a crucial factor to be considered in the treatment of depression.

## Introduction

Comorbid diagnoses are common in mental disorders. For instance, in nationally representative surveys in the United States, Kessler and colleagues found 60% of participants with lifetime Major Depressive Disorder (MDD) to meet criteria of a comorbid anxiety disorder^1^, and, more specifically, about one third suffered from comorbid social phobia^2^. Brown et al.^3^ replicated these findings, which gave rise to future research investigating the potentially similar underlying mechanisms. Repetitive negative thought—such as rumination—seems to be a transdiagnostic factor, which has been shown to be related to the development of both depression and anxiety disorders^4–6^.

Rumination is defined as a perseverative, highly self-referential, pessimistic and abstract thinking style with little or no goal and change-orientation^7^. Furthermore, rumination is conceptualized as a form of repetitive negative thinking, such as worry^8^. Originally investigated by Nolen-Hoeksema under the term “depressive rumination”^9^, it was described as “behavior and thoughts that focus one’s attention on one’s depressive symptoms and on the implications of these symptoms” (p. 569, ^10^). Since then, different models have been established and broadened the conceptualization of rumination. In the investigation of depressive rumination, experimental designs often use instructions to think a certain way, for instance about negative emotions/negative personality attributes^11–13^ or negative life events^14–16^ to induce ruminative processes. However, these approaches, even though they assess self-referential thoughts, which are a typical content of depressive rumination, do not capture other aspects of rumination like uncontrollability or repetitiveness. Indirect induction methods using social stress tests like the Trier Social Stress Test (TSST)^17^ have been able to successfully provoke increased state rumination^18–24^. In line with longitudinal studies, stress-reactive rumination promises to be a better predictor of depression than trait rumination^25^. Interestingly, this is not in line with the original definition of rumination by Nolen-Hoeksema^9^, which assumed ruminative processes to pose more of a trait rather than a reactive state condition^4^. Supporting this assumption of a trait as well as a state pattern of rumination, relatively unstable processes fulfilling the criteria of rumination were found^26–28^. Robinson and Alloy^25^ postulated the Theory of Stress-Reactive Rumination, defining rumination as a state. Taken together, this state rumination pattern delivers an enriching amplification to the original model of trait rumination by Nolen-Hoeksema^4^.

Social anxiety research has to face similar problems in the investigation of post-event processing, which can be defined “as an individual’s repeated consideration and potential reconstruction of his performance following a social situation” (p. 891^29^) and which is postulated to be a maintaining factor in social anxiety disorders^30^. However, studies in which participants are instructed to imagine hypothetical or recent personal situations^31–33^ as well as experimental studies in which impromptu-speech tasks are used^34,35^ show highly socially anxious individuals to be more prone to engage in post-event processing. These effects were maintained even one week after experimentally induced confrontations^36^, as well as in naturalistic reports of diary-based studies^37^. When stressor-induced ruminative processes are compared in depressed patients and socially anxious individuals, studies found the latter to be more strongly^38^ and both uniquely associated with negative post-event rumination^35^. However, it remains unclear in how far momentary ruminative responses represent a construct associated with depressive rumination as defined by Nolen-Hoeksema^9^ and/or social anxiety. Potentially, rumination in depression and post-event-processing in social anxiety are to some extent equivalent constructs, which poses the existence of a common underlying mechanism and transdiagnostic phenomenon.

The Trier Social Stress Test (TSST) is a commonly used, highly potent stressor and the gold standard for examining neurocognitive mechanisms of acute stress^39^. Using the TSST in adults with social phobia, Condren et al.^40^ observed significantly higher stress-induced cortisol increases in patients compared to a control group, suggesting that patients with social phobia are hyper-responsive when confronted with psychological stress^41,42^. Concerning rumination in general, it could be demonstrated that in subjects with higher trait rumination the stress response after a TSST was either intensified or prolonged^23,43,44^. However, there are also studies which could not find any differences of socially phobic individuals compared to healthy controls^45^, or only subjective but not physiological stress responses to be elevated in the former^46^. Investigating the neural correlates of social anxiety in a public speech task, Tuscan et al.^47^ found elevated frontal cortical oxygenation using fNIRS, but no significant differences between high and low socially anxious individuals. Studies using the TSST in a sample of depressed patients revealed hypoactivity in the prefrontal cortex as well as increased state rumination and negative affect when compared to healthy controls^22,48,49^. Generally, increased activity in regions of the Cognitive Control Network (CCN), comprising the dorsolateral prefrontal cortex (DLPFC), anterior cingulate cortex (ACC) and posterior parietal cortex^50^, was observed in fNIRS and fMRI studies as a correlate of social stress induction^51^. Furthermore, in one of our previous studies^22,48^ we found decreased activity in the bilateral inferior frontal gyrus (IFG) as a mediator of post-stress rumination. As the IFG is relevant in inhibition and attention control tasks^51–58^, this finding let us conclude that inhibition of the IFG and therefore of these tasks might lead to rumination. However, up to date, neurocognitive studies investigating the association of stress-reactive rumination with social anxiety and trait rumination are scarce. Following the assumption of a possible common, transdiagnostic factor of rumination in depression and social anxiety, a neurocognitive investigation of stress-reactive rumination in depressed as well as anxious subjects seems to be useful in order to unravel a potentially common ground on a neuronal level.

In two previous studies of our lab^22,48^, we investigated stress-induced rumination but did not test whether social anxiety has an influence or explains unique variance of the found effects. More specifically, we investigated the efficacy of a socially stressful situation (the TSST) under laboratory conditions as well as its effects on post-event processing on a neuronal level to find possible neuronal activation patterns for rumination in different sub-samples (high vs. low trait-ruminators, depressed vs. healthy subjects). Furthermore, we measured the physiological and the affective adaptation to this high-stress situation. Therefore, we assessed behavioral data such as subjective stress, state rumination and mood changes as well as heart rate, salivary cortisol and cortical oxygenation in both studies. In the present analysis, merging and reanalyzing the data of these two studies that had the same procedure, we expected higher subjective stress and increased state rumination and negative mood in socially anxious subjects. On a neural level, we expected that social anxiety would have a negative effect on CCN functioning, as already shown for trait rumination. Furthermore, we hypothesized elevated salivary cortisol levels as well as cortical oxygenation in both, socially anxious subjects as well as high trait ruminators. Generally, in line with research suggesting common underlying mechanisms of social anxiety and ruminative processes, we expected high correlations of the Liebowitz Social Anxiety Scale (LSAS) and the Rumination Response Scale (RRS) but unique variance to be explained by both factors in our corresponding outcome measures.

## Results

### Subjective stress (VAS ratings)

**Figure 1:**
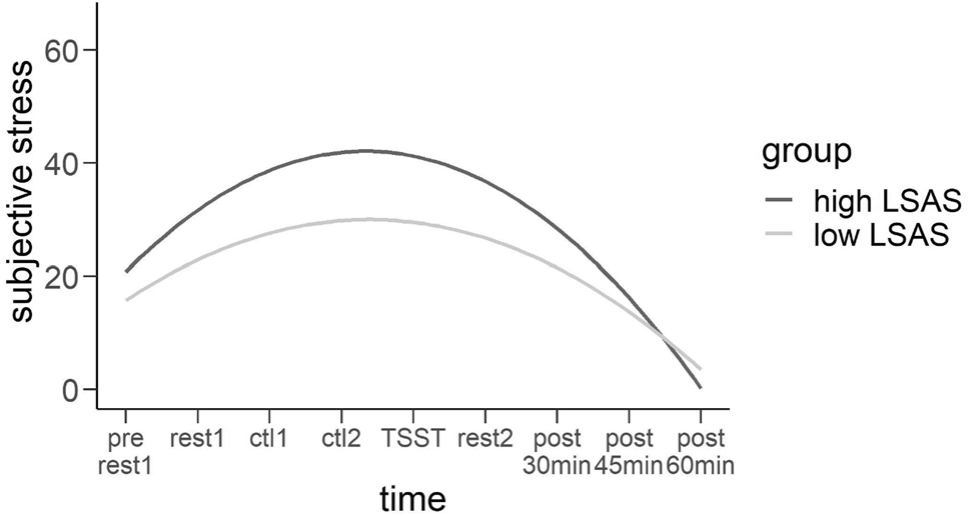
Predicted subjective stress ratings (VAS) in percent dependent on time according to the estimated parameters of the regression model including LSAS (rest1 = resting-state measurement 1, ctl1 = control task 1, ctl2 = control task 2, TSST = stress induction, rest2 = resting-state measurement 2). The significant interaction effect of LSAS and time has been categorized into low and high total scores (± 1 SD) for reasons of clearer visualization.

First, we wanted to investigate whether the stress induction was reflected by changes in subjective stress. Calculating Mahalanobis distances, we identified seven subjects´ stress ratings as multivariate outliers (*p* < 0.001) and therefore excluded them from the following analyses. From previous investigations of stress paradigms^48,49^ we assumed a quadratic relationship of subjective stress ratings and time and therefore tested a linear model against a quadratic model using a Likelihood-ratio-test. Results revealed a significantly better fit of the quadratic model, ${\chi }^{2}$(1) = 244.58, *p* < 0.001, which was subsequently used in the analysis. Fitting the basic model, time yielded a significant predictor as a linear (*t*(664) = 6.607, *β* = 13.395, *p* < 0.001) as well as a quadratic term (*t*(665) = − 2.512, $\beta$ = − 1.538, *p* < 0.05) (see Table 1) and remained significant even in the more complex models. In model 2, we observed significant interactions of LSAS with both time factors linear: (*t*(664) = 2.771, *β* = 2.868, *p* < 0.01; quadratic: *t*(664) = − 2.719, *β* = − 0.339, *p* < 0.01) and more variance to be explained compared to the basic model (change of *R*^2^ = 0.069). Higher LSAS scores were associated with higher linear increases in the stress response and a stronger quadratic decrease post stress (see Fig. 1). In model 3, RRS did not explain further variance (change of R^2^ compared to the basic model = 0.021). In model 4, we observed the same pattern as before whereby both interactions of LSAS and time got significant linear: (*t*:(664) = 2.771, *β* = 2.868, *p* < 0.01 ; quadratic: *t*(664) = − 2.719, $\beta$ = − 0.338, *p* < 0.01) and no change in *R*^2^ was observed compared to model 2. Fitting model 5, only the control variable LSAS yielded a significant predictor (*t*(83) = 4.054$,$ *β* = 6.416, *p* < 0.001). We found the fixed effects of the latter model to explain more variance compared to model 3 (change of *R*^2^ = 0.044). Higher social anxiety was associated with increased subjective stress. The results of the models 1 to 5 are to be found in Table 1. Calculating the most complex model with all interactions (see Table 2), we found the same results as before in model 4 with both time factors as main effects (linear: *t*(664) = 6.782, $\beta$ = 14.171, *p* < 0.001; quadratic: *t*(664) = − 2.654, $\beta$ = − 1.624, *p* < 0.01) as well as their interactions with LSAS (linear: *t*(664) = 2.501, $\beta$ = 3.157, *p* < 0.05; quadratic: *t*(664) = − 2.494, $\beta$ = − 0.379, *p* < 0.05) reaching significance. Model 6 didn’t explain much more variance compared to model 4 (change of *R*^2^ = 0.001) or to model 5 (change of *R*^2^ = 0.005).

**Table 1:** Results of the Mixed Models exploring the association between subjective stress, negative affect, state rumination and social anxiety (LSAS) and trait rumination (RRS) (*AIC* Akaike Information Criterion, *BIC* Bayesian-Information-Criterion, *R*^*2*^ variance explained by the fixed effects). Significant results are bold. *p < 0.05, **p < 0.01, ***p < 0.01. Beta-estimates and standard errors in brackets.

Dependent variables	Model 1: Basic model			Model 2: Basic model + LSAS			Model 3: Basic model + RRS			Model 4: Basic model + LSAS while correcting for RRS			Model 5: Basic Model + RRS while correcting for LSAS		
	VAS	Negative affect	State rumination	VAS	Negative affect	State rumination	VAS	Negative affect	State rumination	VAS	Negative affect	State rumination	VAS	Negative affect	state rumination
Intercept	5.692** (2.132)	21.779*** (0.823)	1.574*** (0.086)	6.082** (2.044)	22.001*** (0.715)	1.582*** (0.068)	5.797** (2.105)	21.774*** (0.738)	1.579*** (0.068)	6.087** (2.043)	21.960*** (0.692)	1.583*** (0.062)	6.423** (2.041)	21.937*** (0.692)	1.584*** (0.061)
Time	13.395*** (2.027)	− 0.139*** (0.032)	0.470*** (0.070)	13.769*** (2.02)	− 0.138*** (0.032)	0.476*** (0.065)	2.495*** (2.025)	− 0.142*** (0.032)	0.473*** (0.065)	13.769*** (2.02)	− 0.140*** (0.032)	0.476*** (0.065)	13.495*** (2.025)	− 0.141*** (0.032)	0.473*** (0.065)
Time^2^	− 1.538* (0.612)	− 0.029*** **(0.003)**		− 1.582** (0.609)	− 0.029*** (0.003)		− 1.549* (0.611)	− 0.029*** (0.003)		− 1.582** (0.609)	− 0.029*** (0.003)		− 1.549* (0.611)	− 0.029*** (0.003)	
Time × time^2^	− 0.027 (0.05)	0.001*** (0.000)		− 0.027 (0.05)	0.001*** (0.000)		− 0.027 (0.05)	0.001*** (0.000)		− 0.027 (0.05)	0.001*** (0.000)		− 0.027 (0.05)	0.001*** (0.000)	
LSAS				2.987 (2.137)	4.365*** (0.716)	0.358*** (0.069)				2.615 (2.265)	3.285*** (0.793)	0.199** (0.070)	6.416*** (1.583)	3.046*** (0.755)	0.332*** (0.062)
Time × LSAS				2.868** (1.035)	0.004 (0.026)	0.267*** (0.067)				2.868** (1.035)	0.004 (0.026)	0.267*** (0.069)			
Time^2^ × LSAS				− 0.339** (0.125)	− 0.000 (0.00)					− 0.338** (0.125)	− 0.000 (0.001)				
RRS							1.343 (1.974)	3.463*** (0.687)	0.340*** (0.069)	0.692 (1.401)	1.937** (0.708)	0.307*** (0.061)	− 1.359 (2.01)	2.002** (0.730)	0.174* (0.069)
Time × RRS							1.284 (0.921)	− 0.000 (0.023)	0.266*** (0.066)				1.284 (0.921)	0.001 (0.023)	0.266*** (0.066)
Time^2^ × RRS							− 0.136 (0.111)	− 0.000 (0.000)					− 0.136 (0.111)	− 0.000 (0.000)	
AIC	6516.1	1639.9	386.79	6493.5	1612.8	326.87	6513.9	1622.3	329.05	6495.3	1607.7	306.53	6500.9	1609.4	306.11
BIC	6543.8	1661.2	399.52	6535.1	1644.8	345.96	6555.4	1654.3	348.14	6541.5	1643.2	328.80	6547.0	1644.9	328.38
R^2^	0.223	0.129	0.078	0.292	0.351	0.428	0.244	0.301	0.416	0.292	0.391	0.526	0.288	0.390	0.526

**Table 2:** Results of the exploratory Mixed Model 6 exploring the association between subjective stress, negative affect, state rumination and social anxiety (LSAS) and trait rumination (RRS) (*AIC* Akaike Information Criterion, *BIC* Bayesian-Information-Criterion, *R*^*2*^ variance explained by the fixed effects). Significant results are bold. *p < 0.05, **p < 0.01, ***p  < 0.001. Beta-estimates and standard errors in brackets.

Dependent variables	Model 6: Interaction model		
	VAS	Negative affect	State rumination
Intercept	5.492* (2.325)	20.857*** (0.795)	1.800*** (0.059)
Time	14.171*** (2.089)	− 0.145*** (0.035)	− 0.240*** (0.036)
Time^2^	− 1.624** (0.612)	− 0.029*** (0.003)	
LSAS	2.425 (2.605)	2.067* (0.917)	0.309*** (0.070)
RRS	0.527 (2.288)	2.554** (0.769)	0.317*** (0.062)
Time × time^2^	− 0.027 (0.050)	0.001*** (0.000)	
Time × LSAS	3.157* (1.262)	− 0.004 (0.036)	− 0.090* (0.042)
Time^2^ × LSAS	− 0.379* (0.152)	− 0.0001 (0.001)	
Time × RRS	− 0.185 (1.109)	0.006 (0.029)	− 0.088* (0.038)
Time^2^ × RRS	0.038 (0.133)	− 0.0001 (0.001)	
LSAS × RRS	1.360 (2.560)	2.132* (0.817)	0.042 (0.057)
Time × LSAS × RRS	− 0.924 (1.240)	0.012 (0.034)	0.005 (0.035)
Time^2^ × LSAS × RRS	0.097 (0.149)	− 0.001 (0.001)	
AIC	6504.3	1609.8	306.3
BIC	6573.6	1663.1	338.2
R^2^	0.293	0.421	0.536

### Negative affect (PANAS)

To investigate changes in negative affect due to the stress induction, we performed a repeated measurements ANOVA with the factor time (pre stress, post stress, post rest2). Note that the third PANAS assessment (post rest2) was different in study 1 (15 min after the stress induction) and study 2 (60 min after the stress induction), which is why we calculated the time in minutes rather than entering time as a factor. One subject from study 1 was excluded from further analyses due to missing values for negative affect and further three subjects in study 2 were excluded as they were multivariate outliers.

Fitting mixed models for negative affect, the basic model including only time as a linear and quadratic term as well as their interaction as predictors significantly predicted changes in negative mood (time: *t*(180.06) = − 4.379, *β* = 21.779, *p* < 0.001, time^2^: *t*(174.60) = − 11.381, *β* = − 0.029, *p* < 0.001, time × time^2^: *t*(177.08) = 10.545, *β* = 0.001, *p* < 0.001). In model 2, LSAS predicted the outcome significantly as a main effect (*t*(107.30) = 6.094, *β* = 3.463, *p* < 0.001). This model explained more variance than model 1 (change of *R*^2^ = 0.222). In model 3, RRS yielded a significant predictor as a main effect (*t*(100.42) = 5.042, *β* = 3.463, *p* < 0.001). Again, this model did explain more variance than model 1 (change of *R*^*2*^ = 0.172). In model 4, LSAS and RRS yielded significant predictors (LSAS: *t*(101.96) = 4.141, *β* = 3.285, *p* < 0.001, RRS: *t*(85.12) = 2.736, *β* = 1.937, *p* < 0.01). Like that, this model explained further variance regarding the fixed effects when compared with model 2 (change of *R*^2^ = 0.04). When fitting model 5 again, RRS significantly predicted the outcome (*t*(96.50) = 2.743, *β* = 2.002, *p* < 0.01), as well as LSAS (*t*(85.76) = 4.032, *β* = 3.046, *p* < 0.001). Compared to model 3, model 5 explained only little further variance (change of *R*^2^ = 0.089) (see Table 1). Fitting model 6 (see Table 2), including all possible interactions, the pattern remains the same as before, even the significant main effect for LSAS is less pronounced (*t*(113) = 2.258, *β* = 2.067, *p* < 0.05). However, the interaction of these two also reached significance (*t*(126.4) = 2.609, *β* = 2.132, *p* < 0.05). This model explained only little further variance than the next fewer complex models 4 (change of *R*^2^ = 0.030) and 5 (change of *R*^2^ = 0.031). Rerunning our analyses with inclusion of multivariate outliers, we found the same as the aforementioned results.

### State rumination

**Figure 2:**
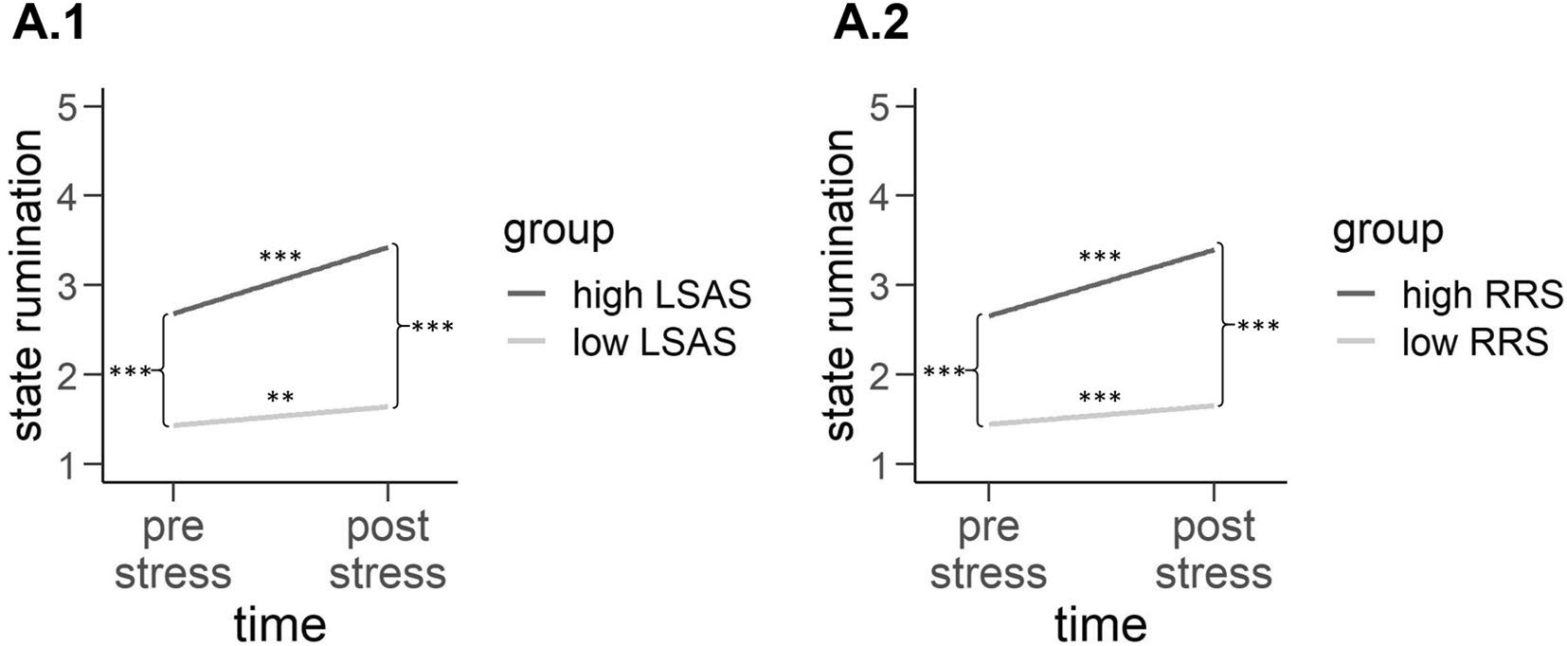
Predicted state rumination dependent on time (pre-stress vs. post-stress) according to the regression model including LSAS (**A.1**) and RRS (**A.2**), respectively. The significant interaction effects of LSAS and time (**A.1**) and RRS and time (**A.2**) have been categorized into low and high total scores (± 1 SD) for reasons of clearer visualization. **p < 0.01; ***p < 0.001.

After exclusion of one subject which yielded a multivariate outlier (*p* < 0.001), we fitted a basic model including the factor time, which predicted the outcome significantly (*t*(89) = 6.63, *β* = 0.470, *p* < 0.001) and remained significant even in the more complex models (see Table 1). Adding LSAS to the model increased the explained variance by 35%. In model 2, the main effect of LSAS (*t*(138.72) = 5.175, *β* = 0.358, *p* < 0.001) as well as the interaction of LSAS and time (*t*(89) = 3.986, *β* = 0.267, *p* < 0.001) significantly predicted state rumination (change in *R*^2^ = 0.35) (see Fig. 2). By fitting model 3, we observed the same effect: RRS (*t*(137.33) = 4.954, *β* = 0.340, *p* < 0.001) as well as the interaction of RRS and time significantly predicted the outcome (*t*(89.00) = 4.048, *β* = 0.266, *p* < 0.001) with additional 33.8% of variance explained (see Fig. 2). Correcting with the corresponding other factor, all predictors of the model significantly predicted the outcome: In case of model 4, time (*t*(89) = 7.289, *β* = 0.476, *p* < 0.001), LSAS (*t*(136.78) = 2.828, *β* = 0.199, *p* < 0.01) and RRS (*t*(89) = 5.040, *β* = 0.307, *p* < 0.001), as well as the interaction of LSAS and time (*t*(89) = 3.986, $\beta$ = 0.267, *p* < 0.001) significantly predicted the outcome. Fixed effects of this model also explained further variance compared to the model only including LSAS (change of *R*^2^ = 0.098). In the case of model 5, also, time (*t*(89) = 7.266, *β* = 0.473, *p* < 0.001), RRS (*t*(136.59) = 2.519, *β* = 0.174, *p* < 0.05), LSAS (*t*(89) = 5.365, *β* = 0.332, *p* < 0.001) and the interaction of RRS and time (*t*(89) = 4.048, *β* = 0.266, *p* < 0.001) significantly predicted the outcome. When comparing the variance explained by the fixed effects of model 3 and model 5, the latter model did explain further variance (change in *R*^2^ = 0.11). Fitting the most complex model, we found a similar pattern of results with strong main effects of time (*t*(89) = − 6.646, *β* = − 0.240, *p* < 0.001), LSAS (*t*(89) = 4.429, *β* = 0.309, *p* < 0.001) and RRS (*t*(89) = 5.094, *β* = 0.317, *p* < 0.001), and less pronounced but still significant interactions of time with LSAS (*t*(89) = − 2.115, *β* = − 0.090, *p* < 0.001) as well as with RRS (*t*(89) = − 2.327, *β* = − 0.088, *p* < 0.001). Compared to models 4 and 5, model 6 explained only little further variance (change of *R*^2^ = 0.010) (see Table 2). Generally, we observed increased state rumination following the TSST in socially anxious individuals as well as individuals with higher trait rumination. Both, trait rumination and social anxiety were uniquely associated with increased state rumination following the TSST. Note that our analyses with inclusion of multivariate outliers yielded the same results.

### fNIRS data

As a next step, we analyzed the association of cortical oxygenation with social anxiety and trait rumination by fitting multiple mixed models with increasing complexity (see Table 3, models 7–12). In each model, we observed a significant main effect for time (model 7: *t*(1260) = 8.693, *β* = 0.143, *p* < 0.001) as well as a marginally significant main effect for the right dorsolateral prefrontal cortex (DLPFC) (model 7: *t*(1260) = 1.837, *β* = 0.078, *p* < 0.1). Furthermore, in all models except for model 12, we found another main effect for the left inferior frontal gyrus (IFG) (model 7: *t*(1260) = − 2.366, *β* = − 0.101, *p* < 0.05).

**Table 3:** Results of the Mixed Models exploring the association between cortical oxygenation in the different ROIs, social anxiety (LSAS) and trait rumination (RRS) (*AIC* Akaike Information Criterion, *BIC* Bayesian-Information-Criterion, *R*^*2*^ variance explained by the fixed effects). Significant results are bold. ^#^ *p *< 0.1, *p< 0.05, **p< 0.01, ***p < 0.001. Beta-estimates and standard errors in brackets.

Dependent variables	Cortical oxygenation					
	Model 7: ROI × time	Model 8: ROI × time × SAS	Model 9: ROI × time × RRS	Model 10: RRS + ROI × time × LSAS	Model 11: LSAS + ROI × time × RRS	Model 12: Interaction model including ROI
Intercept	0.160*** (0.037)	0.160*** (0.036)	0.160*** (0.037)	0.160*** (0.036)	0.160*** (0.036)	0.130** (0.041)
Time	0.143*** (0.016)	0.143*** (0.016)	0.143*** (0.016)	0.143*** (0.016)	0.143*** (0.016)	0.149*** (0.019)
LSAS		− 0.043 (0.037)		− 0.031 (0.042)	− 0.060 (0.038)	**− 0.086**^**#**^** (0.048)**
RRS			− 0.010 (0.037)	− 0.023 (0.038)	0.021 (0.041)	0.031 (0.043)
lIFG	− 0.101* (0.043)	− 0.101* (0.042)	− 0.101* (0.042)	− 0.101* (0.042)	− 0.101* (0.042)	− 0.095* (0.048)
rIFG	− 0.027 (0.043)	− 0.027 (0.042)	− 0.027 (0.042)	− 0.027 (0.042)	− 0.027 (0.042)	0.013 (0.048)
lDLPFC	− 0.018 (0.043)	− 0.018 (0.042)	− 0.018 (0.042)	− 0.018 (0.042)	− 0.018 (0.042)	− 0.036 (0.048)
rDLPFC	**0.078**^**#**^** (0.043)**	**0.078**^**#**^** (0.042)**	**0.078**^**#**^** (0.042)**	**0.078**^**#**^** (0.042)**	**0.078**^**#**^** (0.042)**	0.076 (0.048)
LSAS × RRS						0.059 (0.040)
Time × LSAS		**− 0.028**^**#**^** (0.017)**		**− 0.028**^**#**^** (0.017)**		− 0.002 (0.022)
Time × RRS			− 0.044** (0.016)		− 0.044** (0.016)	− 0.042* (0.020)
Time × LSAS × RRS						− 0.010 (0.018)
lIFG × time	0.009 (0.033)	0.009 (0.033)	0.009 (0.033)	0.009 (0.033)	0.009 (0.033)	0.008 (0.037)
lIFG × LSAS		0.010 (0.043)		0.010 (0.043)		0.039 (0.056)
lIFG × RRS			− 0.027 (0.042)		− 0.027 (0.042)	− 0.046 (0.051)
lIFG × time × LSAS × RRS						0.003 (0.036)
lIFG × time × LSAS		− 0.040 (0.033)		− 0.040 (0.033)		− 0.037 (0.044)
lIFG × time × RRS			− 0.026 (0.033)		− 0.026 (0.033)	− 0.006 (0.039)
lIFG × LSAS × RRS						− 0.011 (0.046)
rIFG × time	− 0.072* (0.033)	− 0.072* (0.033)	− 0.072* (0.033)	− 0.072* (0.033)	− 0.072* (0.033)	− 0.107** (0.037)
rIFG × LSAS		− 0.022 (0.043)		− 0.022 (0.043)		0.015 (0.056)
rIFG × RRS			0.001 (0.042)		0.001 (0.042)	− 0.002 (0.051)
rIFG × time × LSAS		− 0.003 (0.033)		− 0.003 (0.033)		− 0.017 (0.044)
rIFG × time × RRS			− 0.038 (0.033)		− 0.038 (0.033)	− 0.034 (0.039)
rIFG × LSAS × RRS						**− 0.077**^**#**^** (0.046)**
rIFG × time × LSAS × RRS						**0.068**^**#**^** (0.036)**
lDLPFC × time	0.034 (0.033)	0.034 (0.033)	0.034 (0.033)	0.034 (0.033)	0.034 (0.033)	0.057 (0.037)
lDLPFC × LSAS		0.058 (0.043)		0.058 (0.043)		0.062 (0.056)
lDLPFC × RRS			− 0.005 (0.042)		− 0.005 (0.042)	− 0.039 (0.051)
lDLPFC × time × LSAS		− 0.016 (0.033)		− 0.016 (0.033)		− 0.023 (0.044)
lDLPFC × time × RRS			0.004 (0.033)		0.040 (0.033)	0.055 (0.039)
lDLPFC × LSAS × RRS						0.036 (0.046)
lDLPFC × time × LSAS × RRS						− 0.046 8.036)
rDLPFC × time	0.017 (0.033)	0.017 (0.033)	0.017 (0.033)	0.017 (0.033)	0.017 (0.033)	0.009 (0.037)
rDLPFC × LSAS		− 0.006 (0.043)		− 0.006 (0.043)		− 0.001 (0.056)
rDLPFC × RRS			− 0.013 (0.042)		− 0.013 (0.042)	− 0.013 (0.051)
rDLPFC × time × LSAS		0.009 (0.033)		0.009 (0.033)		− 0.002 (0.044)
rDLPFC × time × RRS			0.008 (0.033)		0.008 (0.033)	0.008 (0.039)
rDLPFC × LSAS × RRS						0.004 (0.046)
rDLPFC × time × LSAS × RRS						0.016 (0.036)
AIC	2115.0	2120.5	2112.5	2122.1	2112.0	2135.8
BIC	2177.5	2235.1	2227.0	2241.9	2231.8	2354.6
R^2^	0.061	0.081	0.080	0.082	0.087	0.099

In the first model (model 7), the interaction of the right IFG and time further yielded significance (*t*(1260) = − 2.193, *β* = − 0.027, *p* < 0.05). This interaction remained significant, even when adding LSAS (model 8: *t*(1260) = − 2.201, *β* = − 0.072, *p* < 0.05) and RRS (model 9: *t*(1260) = − 2.193, *β* = − 2.210, *p* < 0.05). Furthermore, in model 8, the interaction between LSAS and time yielded a marginally significant predictor of cortical oxygenation (*t*(1260) = − 1.724, *β* = − 0.028, *p* < 0.1), whereas the interaction term between RRS and time in model 9 reached significance (*t*(1260) = − 2.656, *β* = − 0.044, *p* < 0.01). Both models only explained slightly more variance than model 7 (change of *R*^2^ in model 8 = 0.02; change of *R*^2^ in model 9 = 0.019). The found pattern of results remained the same when we corrected for the corresponding other questionnaire, respectively: the interaction between the right IFG and time remained significant in model 10 (*t*(1260) = − 2.201, *β* = − 0.072, *p* < 0.05) as well as in model 11 (*t*(1260) = − 2.210, *β* = − 0.072, *p* < 0.05). Again, the interaction between time and LSAS reached marginally significance in model 10 (*t*(1260) = − 1.724, *β* = − 0.028, *p* < 0.1) and the interaction of time and RRS got significant in model 11 (*t*(1260) = − 2.656, *β* = − 0.044, *p* < 0.01). Compared to model 8, model 10 didn’t explain much more variance (change of *R*^2^ = 0.001), as well as model 11 when compared to model 9 (change of *R*^2^ = 0.007). As a last step, we additionally examined the interactions between all variables in model 12. Doing so, we found the significant main and interaction effects from the less complex models still reaching significance (lIFG: *t*(1260) = − 1.965, *β* = − 0.095, *p* < 0.05; time × RRS: *t*(1260) = − 2.143, *β* = − 0.042, *p* < 0.05; rIFG × time: *t*(1260) = − 2.864, *β* = − 0.107, *p* < 0.01). Furthermore, in this model marginally significances were found for the main effect of the LSAS (*t*(1260) = − 1.786, *β* = − 0.086, *p* < 0.1) and for the interactions of trait rumination and social anxiety (RRS and LSAS) with the rIFG (*t*(1260) = − 1.672, *β* = − 0.077, *p* < 0.1) and with the rIFG and time (*t*(1260) = 1.891, *β* = 0.068, *p* < 0.1). Only the before found marginally significant main effect of the rDLPFC no longer got significant (*t*(1260) = 1.575, *β* = 0.076, *p* = 0.115). When compared with the next less complex models, model 12 only explained slightly more variance (change of *R*^2^ to model 10 = 0.017; change of *R*^2^ to model 11 = 0.012). The results of all the fitted mixed models are to be found in Table 3.

## Discussion

This study investigated the association of social anxiety and trait rumination with the stress response as induced via the Trier Social Stress Test (TSST). This is crucial as the TSST has recently been used to elicit state ruminative responses as life stress has been shown to be one trigger of rumination and the latter to be a mediating factor of stress and psychopathology^59,60^. In addition, previous findings suggest that social anxiety and depression appear to be uniquely associated with stress induced ruminative processes, although the underlying mechanisms seem to be of a similar manner^35,38^. We analyzed behavioral, neurobiological and autonomous correlates of trait rumination and social anxiety as predictors of the stress response.

Our results showed the same effects of the general stress response as in previously published studies. Concerning our hypotheses, we found a significant moderating effect of social anxiety on subjective stress levels. Intuitively, subjective stress was elevated in socially anxious individuals as they were confronted with a social situation. After similar baseline stress, we observed higher ratings in socially anxious individuals prior to and immediately after the stress induction. This is in line with assumed states of social anxiety including anticipatory processing and post-event processing^29,33,36,37,61–63^. In line with the previous analyses of this data^22,48^, we did not observe such an interaction for the factor of trait rumination. One possible interpretation of these findings might be that even though the intercorrelation of both, RRS and LSAS, is unsurprisingly very high, trait rumination and social anxiety have different effects on subjective stress. In this regard, rumination does not appear to moderate the subjective stress response as social anxiety does. On the other hand, the link between daily life stress and depression (or depressive symptoms) is mediated by rumination^59,64^ and, in the long run, rumination interacts with stressful life events^60,65^. However, the specific types of perceived stress in a social situation and during ruminative processes as well as possible differences between those types need to be further examined. In particular, it would be promising to investigate these associations in clinical subsamples, especially in patients with social anxiety disorder (SAD) with and without comorbid MDD.

On a neurophysiological level, we observed significant time-dependent changes, namely increases in cortical oxygenation due to the stress induction in the CCN. Most interestingly, we observed a significant interaction of time and RRS, more specifically, reduced increases in prefrontal cortical oxygenation in case of higher RRS scores, even when controlled for social anxiety. We observed similar interactions of time and social anxiety, however, this has to be regarded with caution as the interaction of time and LSAS only reached marginal significance. These results substantiate our hypotheses that trait rumination (as well as social anxiety) separately influences stress processing even on a neurophysiological level. More precisely, high trait ruminators seem to have a decreased cortical activation in prefrontal ROIs while processing stress, which in turn might lead to state rumination and dysfunctional coping strategies^67,68,70^. Overall, these results underline our previous analysis of this data, where we also found a pattern of hypo-activation in the whole prefrontal cortex in subjects with MDD^48^ as well as reduced brain activity in the right IFG in high vs. low trait ruminators^22^ during the arithmetic task of the TSST. In this current study we could show that these already found effects stay stable in a larger sample and when controlling for influences of social anxiety.

As the IFG is part of the cognitive control network (CCN), our results go hand in hand with previous investigations that already showed that stress induction paradigms in general lead to increased activity in the CCN^49,51,66^. Furthermore, the decreased cortical activation in prefrontal areas of highly socially anxious individuals as well as high trait ruminators could be interpreted as a manifestation of deficits in cognitive control and inhibition^4^ as well as attentional processes, which are highly demanded in the adaption during the TSST and blend in well with the known functions of the CCN. For example, during the TSST subjects need to refocus their attention after miscalculations or distractions due to the emotionally non-reactive review board. Such deficits in tasks that require attention switching^67,68^, cognitive and attentional control^69^ with attentional biases towards negative information^70^ could already be shown in case of depression and high rumination and it seems conclusive that they might also be apparent in socially anxious individuals, for whom we found similar neural activation patterns. However, these results need to be interpreted with caution as we combined subjects with a current MDD and mentally healthy subjects with a high or a low habit of rumination for our conducted analysis. Even though we already found similar patterns of neuro-activation in separate analyses for each of the subsamples^22,48^, we cannot differentiate the effect of depression on the above findings without any doubt. Therefore, it should be considered that the frontal hemodynamic response and neuronal activation might be reduced in depressive subjects in general, as could be shown in a study by Husain et al.^71^.

On a cognitive and emotional level, the TSST did successfully induce stress-reactive rumination as well as negative affect. We found both measures to be uniquely associated with social anxiety and trait rumination, even when we controlled for different levels of trait rumination and social anxiety, respectively. This is in line with our hypotheses and recent research investigating ruminative responses to stress^20,21,23,24,59,64^ as well as associations of perceived stress in socially anxious individuals^42,72^. Furthermore, considering our exploratory analyses concerning negative affect, highly socially anxious subjects with high trait rumination seem to suffer the most from social stress. As already mentioned, experimental investigations of social stress in socially anxious subjects are scarce. However, it seems to be ostensible that with higher social anxiety there might also be a higher reactivity to socially stressful situations, as indicated by several correlation studies^73–75^.

Intuitively, trait rumination and social anxiety did correlate significantly, which is well in line with the high comorbidity of mood disorders, like MDD, with anxiety disorders. However, our results also show that the previously reported effects of trait rumination on stress-induced processing seem to be independent of the potential confounder of social anxiety, at least to a certain degree. This in turn allows us to deduce some clinical as well as scientific implications: Once more, we could demonstrate that via the TSST state rumination can be reliably induced in high trait ruminators. Moreover, this effect seems to be independent from social anxiety and, therefore, the TSST is a highly efficient tool to induce post-event processing in subjects with and without social anxiety. In a clinical or psychotherapeutic context our results might demonstrate that in the treatment of SAD a training of social skills to handle daily and/or stressful situations will not be sufficient, but it is also important to learn strategies to overcome rumination, for example via emotion regulation strategies^76^.

Additionally, social anxiety and state rumination are not interchangeable as they only explain about 25% of common variance. Supporting this, we found trait rumination and social anxiety to independently influence outcome measures of the TSST on a behavioral as well as on a neurophysiological level. Taken together, these results strengthen the hypothesis of an underlying unifying mechanism which is potentially also transdiagnostic regarding other psychopathologies.

However, some limitations of the study have to be considered when interpreting the results. As not primarily socially anxious participants with diagnosed SAD were recruited, the analyzed data does not cover the full range of variance of social anxiety, especially if it is regarded as a dimensional construct. Even though we did find high variations in LSAS scores, investigations with a clinical sample of patients with SAD would potentially extend evidence for the unique effects of social anxiety on responses to the TSST. Further, we did not collect new data, as our hypotheses have not been analyzed with the used data yet, neither separately for each study nor for the merged sample. However, by merging the two datasets of our lab, we were able to increase the power of our statistical investigations and gather more evidence for the presence of our reported effects. Concerning the investigation of the neural correlates, we used fNIRS, which is highly useful in naturalistic investigations due to decreased sensitivity to motor artifacts^77–79^, but penetration depth is limited to about 1.5–2 cm of the cortex^80^, which restricts our findings to cortical areas. Nevertheless, fNIRS offers the chance to reliably investigate neurobiological mechanisms in an ecologically valid setting^39^.

## Conclusion

Taken together, stress-reactive ruminative responses seem to be linked to social anxiety and high trait rumination as they increased in those subjects after our induction of social stress. Even though our sample consisted mostly of mentally healthy subjects, ruminative responses might be interpreted as a transdiagnostic and dimensional construct in social anxiety and major depression disorder with respect to the current state of research^81,82^. However, both factors, namely social anxiety and trait rumination, were found to be uniquely associated with stress-reactive rumination, even in the examined mostly healthy subjects. Going further, previous studies could show that rumination also seems to play a role in other psychopathological syndromes associated with problems in emotion regulation (e.g. eating disorders^83^, personality disorders^84^ as well as substance abuse^85^). Future research should therefore extend findings of experimentally stress-induced rumination using samples of various mental disorders, as this promises to give support for a transdiagnostic and emotion-reactive understanding of rumination. This, in turn, could help to improve existing and to develop new psychotherapeutic interventions, of which one possible focus should be the treatment of ruminative thinking. For this purpose, fNIRS is a useful tool as it can be assessed easily in an ecologically valid manner (like we did during the TSST) and it is very helpful to investigate the neurobiological basis of ruminative processes. In the future it could be used in a neurofeedback-design to help patients initially recognizing their ruminative processes and to overcome those. In this context, emotion regulation seems to be a promising strategy, which we are currently examining in a depressive sample.

## Materials and methods

### Participants

Please note that the study sample of the current investigation was originally recruited for two distinct studies with a similar study design but a different target population. In study 1, only healthy control subjects without a history of mental or neurological disorder were recruited after being screened using the Ruminative Response Scale (RRS) (see procedures) and categorized in low (PR > 65, n = 22) and high trait ruminators (PR < 27, n = 23) based on 400 subjects that completed an online assessment. In study 2, 23 healthy controls (not differentiated by RRS score) and 22 patients with a current diagnosis of major depressive disorder (MDD) were recruited. Exclusion criteria for both studies were any disorder affecting the cerebral metabolism, heart rate variability and/or cortisol levels: diabetes mellitus, kidney insufficiency, hypertension, dysrhythmia, Cushing syndrome, substance abuse, adrenal insufficiency, cortisone medication, pacemaker, craniocerebral trauma as well as any medication except for oral contraceptives or antidepressants in case of the MDD group. For the MDD subgroup, further exclusion criteria were any other primary mental disorder except ICD-10 diagnosis F32.x, F34.1 and F33.x (The diagnoses in the patient sample included recurrent Major Depressive Disorder (MDD) (*n* = 15) and first episode MDD (*n* = 7). All patients were currently in a depressed state according to their BDI-II score (*M* = 24.14, SD = 11.85). Furthermore, in the assessed baseline PANAS (before the stress induction), the mean negative effect over all depressive subjects was *M* = 23.5 (SD = 9.420) and therefore much higher than the mean negative affect of all other, healthy subjects (*M* = 13.597, SD = 3.416), whereas the mean positive affect in the depressive sample was *M* = 22.818 (SD = 8.830) and therefore lower than the mean positive affect of the healthy sample (*M* = 29.791, SD = 6.210). Both group differences reached significance (negative affect: *t*(22.839) = − 4.828, *p* < 0.001; positive affect: *t*(28.139) = 3.436, *p* < 0.01). The comorbid diagnoses included somatic symptom disorder (*n* = 2), anxiety disorders (*n* = 2) and personality disorders (*n* = 2). 60% of the patients were currently receiving psychotherapy and 58% antidepressant medication.). Further, suicidality, extraordinarily severe depressive symptoms (BDI-II > 50), deficient emotional stability according to the currently treating psychologist and decompensation under social stress in the past led to exclusion. Participants were recruited via emails and flyers, the MDD subgroup was further recruited at the University Hospital of Tübingen and via ambulant psychotherapists. All procedures were approved by the ethics committee at the University Hospital and University of Tübingen and in line with the Declaration of Helsinki in its latest version. All participants gave their written informed consent prior to data collection.

In this analysis, we combined all participants of both studies in order to achieve a broad distribution of our variables of interest, namely trait rumination (RRS scores) and social anxiety (LSAS scores). In addition, as social anxiety and rumination are dimensional rather than categorical constructs and seem to play a role not just in depressed patients but also in healthy controls^86,87^, we rather prioritized a larger than a homogenous sample (in the sense of e.g., depression status). Merging all participants, the mean age of the total sample was 24.11 years (*SD* = 5.24 years) and 80% of all participants were female.

Table 4 gives an overview of the main outcome measures of our analysis, subdivided into the different study samples.

**Table 4:** Demographic variables of the samples.

Variable	Study 1				Study 2			
	Low trait ruminators (*n* = 22)		High trait ruminators (*n* = 23)		Depressed patients (*n* = 22)		Healthy controls (*n* = 23)	
	*M*	*SD*	*M*	*SD*	*M*	*SD*	*M*	*SD*
Age	22.32	3.88	21.70	2.69	27.14	6.15	25.35	5.75
Percent of female participants	86.4		78.3		77.3		78.3	
BDI-II total score	1.95	2.26	8.57	5.80	24.14	11.85	2.13	1.96
RRS mean	1.54	0.22	2.67	0.17	2.59	0.50	1.73	0.39
LSAS mean	0.47	0.19	0.89	0.50	1.32	0.55	0.51	0.33

*BDI-II* Beck Depression Inventory II^58^, *RRS* Rumination Response Scale^9^, *LSAS* Liebowitz Social Anxiety Scale^86^.

### Procedures

**Figure 3:**
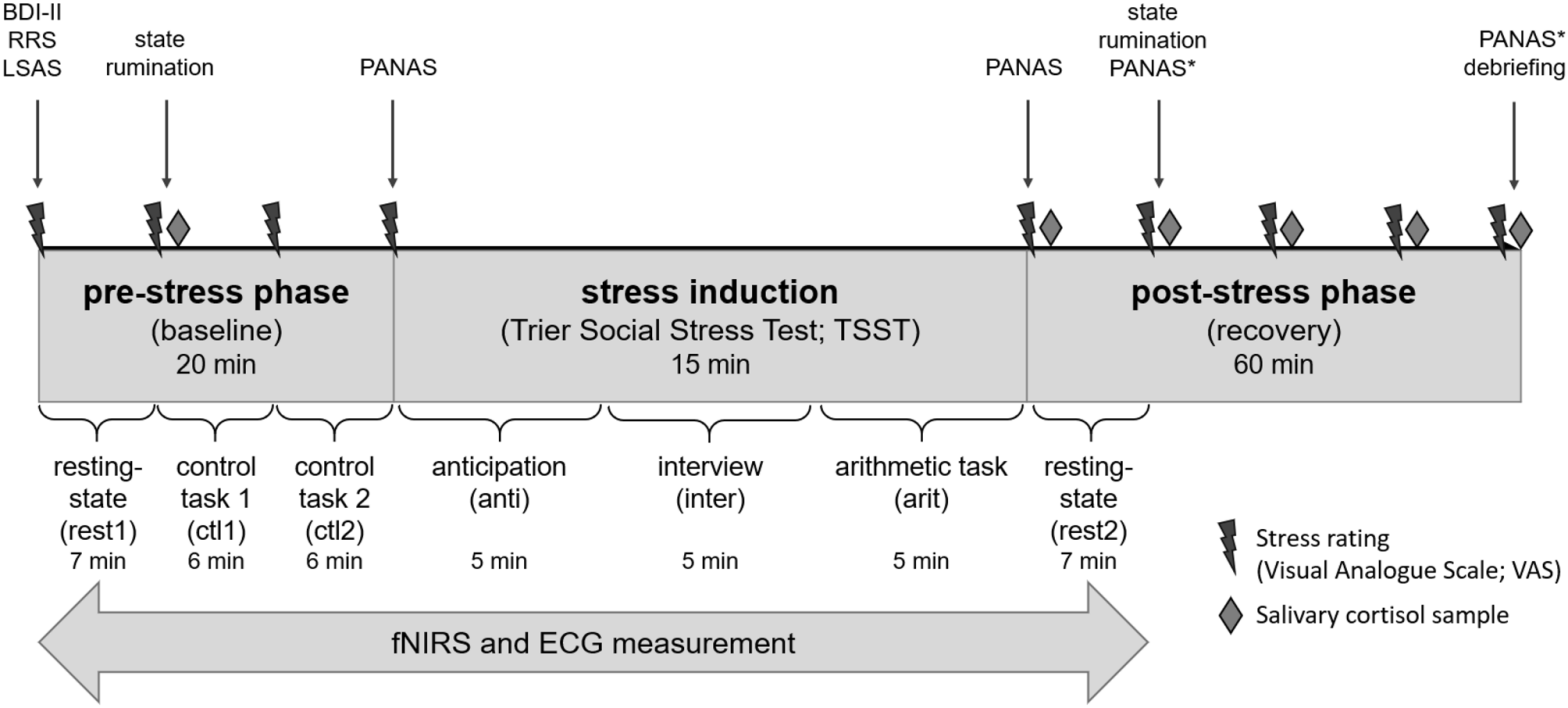
Time course of the TSST. *BDI-II* Beck Depression Inventory II^88^, *RRS* Rumination Response Scale^9^, *LSAS* Liebowitz Social Anxiety Scale^90^, *PANAS* Positive and Negative Affect Scale^97^. *The third PANAS was differently assessed in study 1 (post 15 min) and 2 (post 60 min).

The experimental procure was the same in the case of both studies (see Fig. 3, more detailed information regarding the stress induction and general procedure is to find in^22,48^) and consisted of a pre-stress phase, the stress induction using the Trier Social Stress Test (TSST)^17^ and a post-stress phase during which fNIRS and ECG were assessed. After arriving at the laboratory, participants completed several questionnaires assessing depression symptom severity (BDI-II), trait rumination (RRS), social anxiety symptoms (LSAS) and baseline subjective stress was rated on a Visual Analogue Scale (VAS) ranging from 0 to 100%. This was followed by a 7-min resting-state, another stress rating, a first salivary cortisol sample and two non-stressful control tasks. After each control task, subjective stress was assessed, after the second one also momentary affect (PANAS). Then, the stress induction followed using the TSST which was adapted according to the fNIRS setting: After two experimenters wearing white coats had entered the room, the participant was instructed to prepare for a job interview where they have to give a speech about their strengths and qualifications (anticipation, 5 min). After the preparation, the speech followed (5 min) and an arithmetic task where participants had to subtract 13 from different starting points (6 trials of 40 s calculating and 20 s pause) while holding eye-contact with one of the experimenters and starting all over again in case they made an error. During the stress induction, experimenters remained neutral and socially unresponsive. After the experimenters had left the room, another stress rating and PANAS was assessed as well as another salivary sample. Then, a second resting-state equivalent to the first was performed, state rumination were assessed and every 15 min subjective stress and salivary cortisol were measured.

#### Visual Analogue Scale (VAS)

Throughout the experiment, participants rated their momentary stress levels on a scale ranging from 0 to 100%, where steps of 10% were marked at steps of one centimeter. The questionnaire comprised all ratings of the current day of measurement on one page, so participants were able to allow for their last ratings.

#### Beck Depression Inventory (BDI-II)^88^

In order to screen depression symptom severity, we used the German version of the self-report questionnaire Beck Depression Inventory II. Regarding the previous two weeks, the occurrence of 21 symptoms is rated and symptom severity is assessed as a total score ranging from 0 to 63. Investigating psychometric properties across different populations and languages, respectively, Wang and Gorenstein^89^ could observe overall high internal consistencies (Cronbach’s *α* around 0.9) as well as high retest reliability (mean interval of 2 weeks; *r* around 0.7–0.9).

#### Liebowitz Social Anxiety Scale (LSAS)^90^

The LSAS is a screening measure for social anxiety disorder, comprising 24 social situations that are rated on a 4-point Likert-scale for level of fear (none, mild, moderate, severe) and avoidance (never, occasionally, often, usually) regarding the previous week. The resulting total score (range from 0 to 144) has been shown to have excellent psychometric properties (Cronbach’s *α* = 0.95)^91^. Also, the LSAS was found to be capable of differentiating clinical as well as non-clinical samples^92,93^ which is the reason why in the following we will consider LSAS-scores as a measure of levels of social anxiety. Note that we will report LSAS mean scores in order to account for potential missing values.

#### Ruminative Response Scale (RRS)^9^

Trait rumination was assessed using 22 items which are rated on a 4-point Likert scale ranging from "hardly ever" to "almost always". The total score (range from 22 to 88) has been shown to have high internal consistencies (Cronbach’s α = 0.88–92)^94–96^. Note that we will report RRS mean scores in order to account for potential missing values.

#### Positive and Negative Affect Schedule (PANAS)^97^

Using the PANAS we assessed momentary positive and negative affect. 20 items are rated using 5-point Likert scales ranging from 1 (“very slightly”) to 5 (“extremely”). Both subscales, positive (PA) and negative affect (NA), have acceptable internal consistencies in clinical and non-clinical samples^98,99^ of ɑ = 0.85–0.86 for NA and ɑ = 0.84–0.89 for PA.

#### State rumination

We assessed stress-reactive rumination pre and post stress using adapted items from the RRS^9^, ARSQ^100^, and PTQ^101,102^. Corresponding items were selected according to psychometric evaluations of the authors of those questionnaires. We specifically chose the most selective items of the scales of interest. The eight items were answered using a 5-point Likert scale ranging from 1 (“not at all”) to 5 (“very often”), totaling to a score between 8 and 40. Note that we will report mean scores in order to account for potential missing values. Subjects were instructed to rate whether the items were in line with their mental state during the last 10 min (see Supplementary Table S2).

#### fNIRS

During the stress induction as well as both resting-states, an fNIRS measurement was performed to assess cortical activation using a 46-channel continuous wave multichannel fNIRS system (ETG-4000 Optical Topography System; Hitachi Medical Co., Japan) with a sampling rate of 10 Hz. According to our regions of interest, we placed two frontal probesets and one parietal probeset with reference position to Fpz and Cz according to the 10–20 system^103^ using an Easycap with sponge rings for additional fixation of the optodes (see Supplementary Table S3 and Supplementary Fig. S3). Like this, a fixed inter-optode distance of 3 cm was set for all optodes. Changes in levels of oxygenated (O2Hb) and deoxygenated (HHb) hemoglobin were computed by means of the modified Beer–Lambert Law. Data preprocessing was done in MATLAB^104^, using customized scripts, including interpolation of single noisy channels, correction of motion artifacts using Temporal Derivative Distribution Repair (TDDR) in order to remove spikes primarily caused by head movements in our case^105^ and Correlation-based signal improvement (CBSI)^106^ to combine both signals (O2Hb and HHb) in their merits: High sensibility (O2Hb) and high resilience to arousal artifacts (HHb). We further used bandpass-filtering to remove low-frequency baseline-drifts (below 0.01 Hz) and high-frequency noise (above 0.1 Hz). Then, a second step of channel interpolation followed in case of artifacts due to data correction. Afterwards, a global signal reduction was performed with a spatial gaussian kernel filter with a standard deviation of *σ* = 40^107^. For data analysis, we calculated event-related averages for each trial including a 5 s baseline correction for every region of interest (ROI).

#### Heart rate

We assessed heart rate during both resting-states as well as the stress induction using a 1-channel electrocardiogram in order to monitor heart rate variability as an index of the stress response. After disinfection of the corresponding skin areas, three standard Ag/AgCl ring electrodes were attached using Ten20 conductive paste. Electrodes were placed above the right clavicle, below the left costal arch and on the neck (reference). The signal was recorded using the BrainAmp ExG amplifier with a sampling rate of 1000 Hz and BrainVision Recorder Software (Brain Products, Munich, Germany).

#### Salivary cortisol

Saliva was collected in salivettes (Sarstedt AG & Co., REF 51.1534.500) and was stored at − 20 °C and later thawed and centrifuged for 2 min at 1000*g* to collect saliva. Further analysis was performed with enzyme immunoassay (IBL International, Cortisol ELISA, REF RE52611) according to the manufacturer's instructions. Average cortisol levels were taken from duplicate runs if intra-assay variation was below 10%. Finally, daytime was regressed out of cortisol coefficients to account for circadian rhythm fluctuations that are not related to the TSST and values were log-transformed. Participants were instructed not to drink alcohol the day before the measurement, to sleep as long as they usually do and to perform no physical activities on the day of the measurement. Also, subjects were told not to drink or eat 30 min before the measurement started.

### Data analysis

After data preprocessing, data analysis was done using R^108^ and SPSS^109^. Mixed models were fitted using the R packages lme4^110^ and lmerTest^111^ in order to obtain *p*-values using the Satterthwaite approximation. Graphics were plotted using the R package ggplot2^112^ and MATLAB^104^. Using the R-package MuMIn^113^, marginal *R*^2^ was computed as a measure of variance explained by the fixed effects in the mixed models. By reporting changes in *R*^2^ we are comparing the more complex with the corresponding less complex model.

In this paper, we analyzed subjective stress, negative affect and state rumination. Further, we analyzed heart rate and salivary cortisol, but these analyses are to be found in the supplemental material as they were not related to our primary hypotheses. We investigated in how far social anxiety (LSAS) and trait rumination (RRS) play a role in each measure and set up mixed models with an increasing number of parameters. Note that we did not include BDI-II as a predictor due to its high correlations with social anxiety, *r*(88) = 0.599, *p* < 0.001, and trait rumination, *r*(88) = 0.603, *p* < 0.001, and issues of multicollinearity. Fitting mixed models offers the chance to include random effects accounting for non-independence^114^, as well as to handle unbalanced data and include continuous predictors^115^. First, we conducted a basic model consisting of the parameters set by the experimental design, such as time (model 1). For the two more complex models, we added z-standardized LSAS (model 2) and z-standardized RRS total scores (model 3) as main effects and interaction effects with time, accounting for social anxiety and trait rumination, respectively. We then further added the z-standardized RRS (model 4) and LSAS scores (model 5) only as main effects to the previous models. Like that, we controlled for trait rumination and social anxiety and investigated whether there is unique variance explained by the corresponding measure. Finally, we conducted a model including all possible interactions as most complex model (model 6) to further highlight the possibly different roles of LSAS and RRS in the stress-response. However, this last model is to be seen more as an exploratory analysis due to the problem of collinearity, as the two variables RRS and LSAS are highly correlated (*r* = 0.519***) and therefore an interpretation of the slope coefficients as well as of the changes in one predictor, while holding the other constant, is not easily possible^116–118^. Note that all predictors were included as fixed effects whereas intercepts for every participant were modeled as random effects.

For the analysis of cortical oxygenation, we calculated hierarchical mixed models to investigate the impact of social anxiety (LSAS) and trait rumination (RRS) dependent on ROI and time (control task 1 vs. control task 2 vs. arithmetic task). Therefore, we first conducted a model consisting of time and the different regions of interest (ROI) (model 7). In a next step, we added z-standardized LSAS (model 8) and RRS scores (model 9). In the more complex models, we then included z-standardized LSAS (or RRS), ROI, time as well as their three-way interactions (models 10 and 11). Again, as most complex model, we added all possible interactions (model 12). As before, this model needs to be interpreted with caution due to the problem of collinearity. All aforementioned predictors were included as fixed effects, whereas individual intercepts per participant were modeled as random effects, respectively. Table 5 gives an overview over the models conducted for each measure.

**Table 5:** Parameters included in the mixed models for the outcome measures (models 1–6: subjective stress, negative affect, state rumination; models 7–12: cortical oxygenation).

Name	Parameters
Model 1: Basic model	Time
Model 2: Basic model including LSAS	Time + LSAS + time:LSAS
Model 3: Basic model including RRS	Time + RRS + time:RRS
Model 4: Basic model including LSAS and correcting for RRS	Time + RRS + LSAS + time:LSAS
Model 5: Basic model including RRS and correcting for LSAS	Time + LSAS + RRS + time:RS
Model 6: Interaction model	Time × LSAS × RRS
Model 7: Basic model including time and ROI	ROI:time
Model 8: Basic model including LSAS, time and ROI	ROI:time:LSAS
Model 9: Basic model including RRS, time and ROI	ROI:time:RRS
Model 10: Basic model including LSAS, time, ROI and correcting for RRS	RRS + ROI:time:LSAS
Model 11: Basic model including RRS, time, ROI and correcting for LSAS	LSAS + ROI:time:RRS
Model 12: Interaction model including ROI	Time × LSAS × RRS × ROI

In case of subjective stress, negative affect and heart rate, time was modelled as linear and quadratic term. Colons symbolize interaction effects. All included parameters except for time and region of interest (ROI) were z-standardized. In the models : means interaction effects, whereas × means interaction as well as the corresponding main effects (corresponding to the syntax of R).

Prior to our analyses, we checked assumptions of the used methods and corrected for potential violations of assumptions. Note that corrections were not applied unless stated otherwise. In case of subjective stress ratings and state rumination, normality assumption was not met but due to insufficient success of common transformations like square root, cube root, and log-transformation and the robustness of the mixed models^119^, we decided to perform the analyses as planned. Furthermore, in case of a detection of outliers, we will report the corrected analyses in detail and summarize results of the uncorrected analyses.

Note that physiological data of heart rate was missing in five subjects, leaving 85 participants, and one participant had to be excluded in the analyses of negative affect for study 1, due to missing data.

## Supplementary Information

Supplementary Information.

## Data Availability

The data of this study is available upon request from the first or the last author.

## References

[1] Kessler RC (2003). The epidemiology of major depressive disorder: Results from the National Comorbidity Survey Replication (NCS-R). JAMA.

[2] Kessler RC (1996). Comorbidity of DSM–III–R major depressive disorder in the general population: Results from the US national comorbidity survey. Br. J. Psychiatry.

[3] Brown TA, Campbell LA, Lehman CL, Grisham JR, Mancill RB (2001). Current and lifetime comorbidity of the DSM-IV anxiety and mood disorders in a large clinical sample. J. Abnorm. Psychol..

[4] Smith JM, Alloy LB (2009). A roadmap to rumination: A review of the definition, assessment, and conceptualization of this multifaceted construct. Clin. Psychol. Rev..

[5] Michl LC, McLaughlin KA, Shepherd K, Nolen-Hoeksema S (2013). Rumination as a mechanism linking stressful life events to symptoms of depression and anxiety: Longitudinal evidence in early adolescents and adults. J. Abnorm. Psychol..

[6] Nolen-Hoeksema S (2000). The role of rumination in depressive disorders and mixed anxiety/depressive symptoms. J. Abnorm. Psychol..

[7] Teismann T (2012). Kognitive Verhaltenstherapie depressiven Grübelns.

[8] McEvoy PM, Watson H, Watkins ER, Nathan P (2013). The relationship between worry, rumination, and comorbidity: Evidence for repetitive negative thinking as a transdiagnostic construct. J. Affect. Disord..

[9] Nolen-Hoeksema S (1991). Responses to depression and their effects on the duration of depressive episodes. J. Abnorm. Psychol..

[10] Nolen-Hoeksema S, Morrow J (1991). A prospective study of depression and posttraumatic stress symptoms after a natural disaster: The 1989 Loma Prieta earthquake. J. Pers. Soc. Psychol..

[11] Cooney RE, Joormann J, Eugène F, Dennis EL, Gotlib IH (2010). Neural correlates of rumination in depression. Cogn. Affect. Behav. Neurosci..

[12] Huffziger S, Kuehner C (2009). Rumination, distraction, and mindful self-focus in depressed patients. Behav. Res. Ther..

[13] Watkins E, Brown RG (2002). Rumination and executive function in depression: An experimental study. J. Neurol. Neurosurg. Psychiatry.

[14] Damasio AR (2000). Subcortical and cortical brain activity during the feeling of self-generated emotions. Nat. Neurosci..

[15] Milazzo A-C (2016). Identification of mood-relevant brain connections using a continuous, Subject-driven rumination paradigm. Cereb. Cortex.

[16] Rosenbaum D (2020). Amplitude of low frequency fluctuations (ALFF) of spontaneous and induced rumination in major depression: An fNIRS study. Sci. Rep..

[17] Kirschbaum C, Pirke K-M, Hellhammer DH (1993). The ‘Trier Social Stress Test’—A tool for investigating psychobiological stress responses in a laboratory setting. Neuropsychobiology.

[18] De Witte S (2020). The effect of neurostimulation applied to the left dorsolateral prefrontal cortex on post-stress adaptation as a function of depressive brooding. Prog. Neuropsychopharmacol. Biol. Psychiatry.

[19] Gianferante D (2014). Post-stress rumination predicts HPA axis responses to repeated acute stress. Psychoneuroendocrinology.

[20] Hilt LM, Aldao A, Fischer K (2015). Rumination and multi-modal emotional reactivity. Cogn. Emot..

[21] Kelly MM, Tyrka AR, Anderson GM, Price LH, Carpenter LL (2008). Sex differences in emotional and physiological responses to the Trier Social Stress Test. J. Behav. Ther. Exp. Psychiatry.

[22] Rosenbaum D (2018). Stress-related dysfunction of the right inferior frontal cortex in high ruminators: An fNIRS study. NeuroImage Clin..

[23] Shull A (2016). Trait and state rumination interact to prolong cortisol activation to psychosocial stress in females. Psychoneuroendocrinology.

[24] Zoccola PM, Dickerson SS, Zaldivar FP (2008). Rumination and cortisol responses to laboratory stressors. Psychosom. Med..

[25] Robinson MS, Alloy LB (2003). Negative cognitive styles and stress-reactive rumination interact to predict depression: A prospective study. Cogn. Ther. Res..

[26] Bean CAL, Heggeness LF, Kalmbach DA, Ciesla JA (2020). Ruminative inertia and its association with current severity and lifetime course of depression. Clin. Psychol. Sci..

[27] Everaert J, Joormann J (2020). Emotion regulation habits related to depression: A longitudinal investigation of stability and change in repetitive negative thinking and positive reappraisal. J. Affect. Disord..

[28] Struijs SY (2020). Temporal stability of symptoms of affective disorders, cognitive vulnerability and personality over time. J. Affect. Disord..

[29] Brozovich F, Heimberg RG (2008). An analysis of post-event processing in social anxiety disorder. Clin. Psychol. Rev..

[30] Clark DM, Wells A (1995). A cognitive model of social phobia. Soc. Phob. Diagn. Assess. Treat..

[31] Kocovski NL, Endler NS, Rector NA, Flett GL (2005). Ruminative coping and post-event processing in social anxiety. Behav. Res. Ther..

[32] Kocovski NL, Rector NA (2007). Predictors of post-event rumination related to social anxiety. Cogn. Behav. Ther..

[33] Rachman S, Grüter-Andrew J, Shafran R (2000). Post-event processing in social anxiety. Behav. Res. Ther..

[34] Abbott MJ, Rapee RM (2004). Post-event rumination and negative self-appraisal in social phobia before and after treatment. J. Abnorm. Psychol..

[35] Edwards SL, Rapee RM, Franklin J (2003). Postevent rumination and recall bias for a social performance event in high and low socially anxious individuals. Cogn. Ther. Res..

[36] Dannahy L, Stopa L (2007). Post-event processing in social anxiety. Behav. Res. Ther..

[37] Lundh L-G, Sperling M (2002). Social anxiety and the post-event processing of socially distressing events. Cogn. Behav. Ther..

[38] Kashdan TB, Roberts JE (2007). Social anxiety, depressive symptoms, and post-event rumination: Affective consequences and social contextual influences. J. Anxiety Disord..

[39] Allen AP (2017). The Trier Social Stress Test: Principles and practice. Neurobiol. Stress.

[40] Condren R, O'Neill A, Ryan M, Barrett P, Thakore J (2002). HPA axis response to a psychological stressor in generalised social phobia. Psychoneuroendocrinology.

[41] Furlan PM, DeMartinis N, Schweizer E, Rickels K, Lucki I (2001). Abnormal salivary cortisol levels in social phobic patients in response to acute psychological but not physical stress. Biol. Psychiatry..

[42] Levin AP (1993). Responses of “generalized” and “discrete” social phobics during public speaking. J. Anxiety Disord..

[43] Aldao A, McLaughlin KA, Hatzenbuehler ML, Sheridan MA (2014). The relationship between rumination and affective, cognitive, and physiological responses to stress in adolescents. J. Exp. Psychopathol..

[44] Denson TF, Fabiansson EC, Creswell JD, Pedersen WC (2009). Experimental effects of rumination styles on salivary cortisol responses. Motiv. Emot..

[45] Martel FL (1999). Salivary cortisol levels in socially phobic adolescent girls. Depress. Anxiety.

[46] von Dawans B, Trueg A, Kirschbaum C, Fischbacher U, Heinrichs M (2018). Acute social and physical stress interact to influence social behavior: The role of social anxiety. PLoS One.

[47] Tuscan L-A (2013). Exploring frontal asymmetry using functional near-infrared spectroscopy: A preliminary study of the effects of social anxiety during interaction and performance tasks. Brain Imaging Behav..

[48] Rosenbaum D (2021). Insights from a laboratory and naturalistic investigation on relationships between stress, rumination and frontal brain functioning in MDD: An fNIRS study. Neurobiol. Stress.

[49] Rosenbaum D (2018). Cortical hemodynamic changes during the Trier Social Stress Test: An fNIRS study. Neuroimage.

[50] Niendam TA (2012). Meta-analytic evidence for a superordinate cognitive control network subserving diverse executive functions. Cogn. Affect. Behav. Neurosci..

[51] Kogler L (2015). Psychosocial versus physiological stress—Meta-analyses on deactivations and activations of the neural correlates of stress reactions. Neuroimage.

[52] Aron AR, Monsell S, Sahakian BJ, Robbins TW (2004). A componential analysis of task-switching deficits associated with lesions of left and right frontal cortex. Brain.

[53] Aron AR, Robbins TW, Poldrack RA (2004). Inhibition and the right inferior frontal cortex. Trends Cogn. Sci..

[54] Depue BE, Curran T, Banich MT (2007). Prefrontal regions orchestrate suppression of emotional memories via a two-phase process. Science.

[55] Wang J (2005). Perfusion functional MRI reveals cerebral blood flow pattern under psychological stress. Proc. Natl. Acad. Sci..

[56] Garavan H, Ross TJ, Stein EA (1999). Right hemispheric dominance of inhibitory control: An event-related functional MRI study. Proc. Natl. Acad. Sci. U.S.A..

[57] Konishi S, Nakajima K, Uchida I, Sekihara K, Miyashita Y (1998). No-go dominant brain activity in human inferior prefrontal cortex revealed by functional magnetic resonance imaging: No-go dominant brain activity revealed by fMRI. Eur. J. Neurosci..

[58] Rubia K, Smith AB, Brammer MJ, Taylor E (2003). Right inferior prefrontal cortex mediates response inhibition while mesial prefrontal cortex is responsible for error detection. Neuroimage.

[59] Ruscio AM (2015). Rumination predicts heightened responding to stressful life events in major depressive disorder and generalized anxiety disorder. J. Abnorm. Psychol..

[60] Connolly SL, Alloy LB (2017). Rumination interacts with life stress to predict depressive symptoms: An ecological momentary assessment study. Behav. Res. Ther..

[61] Vassilopoulos. Anticipatory processing plays a role in maintaining social anxiety. *Anxiety Stress Coping.* **18**, 321–332 (2005).

[62] Hinrichsen H, Clark DM (2003). Anticipatory processing in social anxiety: Two pilot studies. J. Behav. Ther. Exp. Psychiatry.

[63] Vassilopoulos. Anticipatory processing in social anxiety. *Behav. Cogn. Psychother.* **32**, 303–311 (2004).

[64] Moberly NJ, Watkins ER (2008). Ruminative self-focus and negative affect: An experience sampling study. J. Abnorm. Psychol..

[65] Kircanski K, Thompson RJ, Sorenson J, Sherdell L, Gotlib IH (2018). The everyday dynamics of rumination and worry: Precipitant events and affective consequences. Cogn. Emot..

[66] Henze GI (2020). Increasing deactivation of limbic structures over psychosocial stress exposure time. Biol. Psychiatry Cogn. Neurosci. Neuroimaging.

[67] Koster EHW, De Lissnyder E, De Raedt R (2013). Rumination is characterized byvalence-specific impairments in switching of attention. Acta Physiol. (Oxf.).

[68] Whitmer AJ, Banich MT (2007). Inhibition versus switching deficits in different forms of rumination. Psychol. Sci..

[69] Ottowitz WE, Tondo L, Dougherty DD, Savage CR (2002). The neural network basis for abnormalities of attention and executive function in major depressive disorder: Implications for application of the medical disease model to psychiatric disorders. Harv. Rev. Psychiatry.

[70] Koster EHW, De Raedt R, Goeleven E, Franck E, Crombez G (2005). Mood-congruent attentional bias in dysphoria: Maintained attention to and impaired disengagement from negative information. Emotion.

[71] Husain FS (2020). Validating a functional near-infrared spectroscopy diagnostic paradigm for Major Depressive Disorder. Sci. Rep..

[72] Klumbies E, Braeuer D, Hoyer J, Kirschbaum C (2014). The reaction to social stress in social phobia: Discordance between physiological and subjective parameters. PLoS One.

[73] Valena SP, Szentagotái-Tatar A (2015). The relationships between stress, negative affect, rumination and social anxiety. J. Evid. Based Psychother..

[74] De Castella K (2014). Emotion beliefs in social anxiety disorder: Associations with stress, anxiety, and well-being. Aust. J. Psychol..

[75] Kropf E, Syan SK, Minuzzi L, Frey BN (2019). From anatomy to function: The role of the somatosensory cortex in emotional regulation. Braz. J. Psychiatry.

[76] Moscovitch DA (2013). Self-portrayal concerns and their relation to safety behaviors and negative affect in social anxiety disorder. Behav. Res. Ther..

[77] Ehlis A-C, Schneider S, Dresler T, Fallgatter AJ (2014). Application of functional near-infrared spectroscopy in psychiatry. Neuroimage.

[78] Hajime, M. In *Application of Near Infrared Spectroscopy in Biomedicine Handbook of Modern Biophysics* (eds Thomas, J. & Kazumi, M.) 59–74 (Springer US, 2013).

[79] Jue, T. & Masuda, K. *Application of Near Infrared Spectroscopy in Biomedicine*. (Springer, 2013).

[80] Haeussinger FB (2011). Simulation of near-infrared light absorption considering individual head and prefrontal cortex anatomy: Implications for optical neuroimaging. PLoS One.

[81] Harvey, A. G., Watkins, E. & Mansell, W. *Cognitive Behavioural Processes Across Psychological Disorders: A Transdiagnostic Approach to Research and Treatment*. (OUP Oxford, 2004).

[82] Klemanski DH, Curtiss J, McLaughlin KA, Nolen-Hoeksema S (2017). Emotion regulation and the transdiagnostic role of repetitive negative thinking in adolescents with social anxiety and depression. Cogn. Ther. Res..

[83] Naumann E, Tuschen-Caffier B, Voderholzer U, Svaldi J (2016). Spontaneous emotion regulation in anorexia and bulimia nervosa. Cogn. Ther. Res..

[84] Meaney R, Hasking P, Reupert A (2016). Borderline personality disorder symptoms in college students: The complex interplay between alexithymia, emotional dysregulation and rumination. PLoS One.

[85] Nolen-Hoeksema S, Stice E, Wade E, Bohon C (2007). Reciprocal relations between rumination and bulimic, substance abuse, and depressive symptoms in female adolescents. J. Abnorm. Psychol..

[86] Lois G, Wessa M (2016). Differential association of default mode network connectivity and rumination in healthy individuals and remitted MDD patients. Soc. Cogn. Affect. Neurosci..

[87] Berman MG (2011). Depression, rumination and the default network. Soc. Cogn. Affect. Neurosci..

[88] Hautzinger, M., Keller, F. & Kühner, C. *BDI-II. Beck-Depressions-Inventar. Revision. 2, Auflage*. (Pearson Assessment, 2009).

[89] Wang Y-P, Gorenstein C (2013). Psychometric properties of the Beck Depression Inventory-II: A comprehensive review. Braz. J. Psychiatry.

[90] Liebowitz, M. R. In *Modern Trends in Pharmacopsychiatry* vol. 22 (ed Klein, D. F.) 141–173 (S. Karger AG, 1987).

[91] Baker SL, Heinrichs N, Kim H-J, Hofmann SG (2002). The Liebowitz social anxiety scale as a self-report instrument: A preliminary psychometric analysis. Behav. Res. Ther..

[92] Heimberg RG, Holaway RM (2007). Examination of the known-groups validity of the Liebowitz Social Anxiety Scale. Depress. Anxiety.

[93] Rytwinski NK (2009). Screening for social anxiety disorder with the self-report version of the Liebowitz Social Anxiety Scale. Depress. Anxiety.

[94] Papageorgiou, C. & Wells, A. *Depressive Rumination: Nature, Theory, and Treatment*. (Wiley, 2004).

[95] Treynor W, Gonzalez R, Nolen-Hoeksema S (2003). Rumination reconsidered: A psychometric analysis. Cogn. Ther. Res..

[96] Just N, Alloy LB (1997). The response styles theory of depression: Tests and an extension of the theory. J. Abnorm. Psychol..

[97] Watson D, Clark LA, Tellegen A (1988). Development and validation of brief measures of positive and negative affect: The PANAS scales. J. Pers. Soc. Psychol..

[98] Crawford JR, Henry JD (2004). The Positive and Negative Affect Schedule (PANAS): Construct validity, measurement properties and normative data in a large non-clinical sample. Br. J. Clin. Psychol..

[99] Krohne HW, Egloff B, Kohlmann C-W, Tausch A (1996). Untersuchungen mit einer deutschen Version der "Positive and Negative Affect Schedule" (PANAS). [Investigations with a German version of the Positive and Negative Affect Schedule (PANAS).]. Diagnostica-Gottingen.

[100] Diaz BA (2013). The Amsterdam Resting-State Questionnaire reveals multiple phenotypes of resting-state cognition. Front. Hum. Neurosci..

[101] de Jong-Meyer R, Parthe T, Projektgruppe (2009). Einfluss von Achtsamkeitsübung und Dezentrierung auf Rumination und Spezifität autobiographischer Erinnerungen. Z. Klin. Psychol. Psychother..

[102] Ehring T (2011). The Perseverative Thinking Questionnaire (PTQ): Validation of a content-independent measure of repetitive negative thinking. J. Behav. Ther. Exp. Psychiatry.

[103] Jasper H (1958). Report of the committee on methods of clinical examination in electroencephalography. Electroencephalogr. Clin. Neurophysiol..

[104] MathWorks Inc (2017). MATLAB R2017a.

[105] Fishburn FA, Ludlum RS, Vaidya CJ, Medvedev AV (2019). Temporal Derivative Distribution Repair (TDDR): A motion correction method for fNIRS. Neuroimage.

[106] Cui X, Bray S, Reiss AL (2010). Functional near infrared spectroscopy (NIRS) signal improvement based on negative correlation between oxygenated and deoxygenated hemoglobin dynamics. Neuroimage.

[107] Zhang X, Noah JA, Hirsch J (2016). Separation of the global and local components in functional near-infrared spectroscopy signals using principal component spatial filtering. Neurophotonics.

[108] R Core Team. *R: A Language and Environment for Statistical Computing (4.0.0) [Computer software]*. (2019).

[109] IBM Corp. *IBM SPSS Statistics for Windows*. 27 ed. (IBM Corp, 2020).

[110] Bates, D., Mächler, M., Bolker, B. & Walker, S. Fitting linear mixed-effects models using lme4. *arXiv preprint arXiv:1406.5823* (2014).

[111] Kuznetsova A, Brockhoff PB, Christensen RH (2017). lmerTest package: Tests in linear mixed effects models. J. Stat. Softw..

[112] Wickham H (2009). Elegant Graphics for Data Analysis.

[113] MuMIn: Multi-Model Inference; R Package Version 1.43. 17; 2020 v. 1.43.17 (2020).

[114] Singmann H, Kellen D (2019). An introduction to mixed models for experimental psychology. New Methods Cogn. Psychol..

[115] Judd CM, Westfall J, Kenny DA (2012). Treating stimuli as a random factor in social psychology: A new and comprehensive solution to a pervasive but largely ignored problem. J. Pers. Soc. Psychol..

[116] Bi J (2012). A review of statistical methods for determination of relative importance of correlated predictors and identification of drivers of consumer liking. J. Sens. Stud..

[117] Feig DG (1978). Ridge regression: When biased estimation is better. Soc. Sci. Q..

[118] Gregorich M, Strohmaier S, Dunkler D, Heinze G (2021). Regression with highly correlated predictors: variable omission is not the solution. Int. J. Environ. Res. Public Health.

[119] Schielzeth H (2020). Robustness of linear mixed-effects models to violations of distributional assumptions. Methods Ecol. Evol..

[120] Cutini S, Scatturin P, Zorzi M (2011). A new method based on ICBM152 head surface for probe placement in multichannel fNIRS. Neuroimage.

