# Supplementary material for: Trait rumination and social anxiety separately influence stress-induced rumination and hemodynamic responses
Source: Sci Rep. 2022 Apr 1;12:5512. doi: 10.1038/s41598-022-08579-1 (PMC8976084; doi:10.1038/s41598-022-08579-1)
Supplement: Supplementary file 1 — Supplementary Information. [file 41598_2022_8579_MOESM1_ESM.pdf]

Supplementary Material: Trait rumination and social anxiety separately influence stress induced rumination and hemodynamic responses

Hendrik Laicher*[1], Isabell Int-Veen*[1,2], Florian Torka[2], Agnes Kroczek[1], Isabel Bihlmaier[1], Helena Storchak[1], Kerstin Velten-Schurian[1], Thomas Dresler[1,3], Ramona Täglich[1], Andreas J. Fallgatter[1,3], Ann-Christine Ehlis[1,3], David Rosenbaum[1]

*The authors wish it to be known that, in their opinion, the first two authors should be regarded as joint first authors.

[1]Department of Psychiatry and Psychotherapy, University Hospital of Tübingen, Tübingen, Germany
[2]Department of Psychology, University of Tübingen, Germany
[3]LEAD Graduate School & Research Network, University of Tübingen, Tübingen, Germany

Corresponding author:

Hendrik Laicher[1]
Department of Psychiatry and Psychotherapy
University Hospital of Tübingen
Calwerstraße 14
72076 Tübingen
Germany

E-mail: hendrik.laicher@med.uni-tuebingen.de
Phone: 0049 7071 29-87103

Supplementary material

Heart rate

To investigate whether the stress induction was reflected by an increase in peripheral-physiological variables, we analyzed the mean heart rate by calculating the beats per minute for each part of the measurement. Calculating the mahalanobis distances, we identified two subjects´ heart rates as multivariate outliers and therefore excluded them in our analyses. With the data of the remaining 83 subjects, we performed a one-way repeated measurements ANOVA for time. Correcting for violated sphericity assumption using Greenhouse-Geisser procedure ($\varepsilon = 0.427$), we found a significant main effect of time, $F(2.56, 210.21) = 157.959$, $p < .001$, $\eta_G^2 = .312$. Planned ongoing contrasts revealed a significant increase in heart rate from the first resting-state measurement (rest1) to the first control task (ctrl1) ($t(82) = -19.124$, $p < .001$, $d = -2.099$), to the second control task (ctrl2) ($t(82) = -3.664$, $p < .001$, $d = -0.402$), a significant decrease from the second control task (ctrl2) to the anticipation phase (anti) ($t(82) = 4.861$, $p < .001$, $d = 0.534$), again a significant increase from the anticipation phase (anti) to the job-interview (inter) ($t(82) = -13.256$, $p < .001$, $d = -1.455$), a significant decrease from the interview to the arithmetic task (arit) ($t(82) = 3.299$, $p < .01$, $d = 0.362$) as well as a significant decrease from the arithmetic task to the second resting-state measurement (rest2) ($t(82) = 19.690$, $p < .001$, $d = 2.161$) (see figure S1). Rerunning our analysis with inclusion of the outlier, we do no longer find a significant difference between the job interview and the arithmetic task.

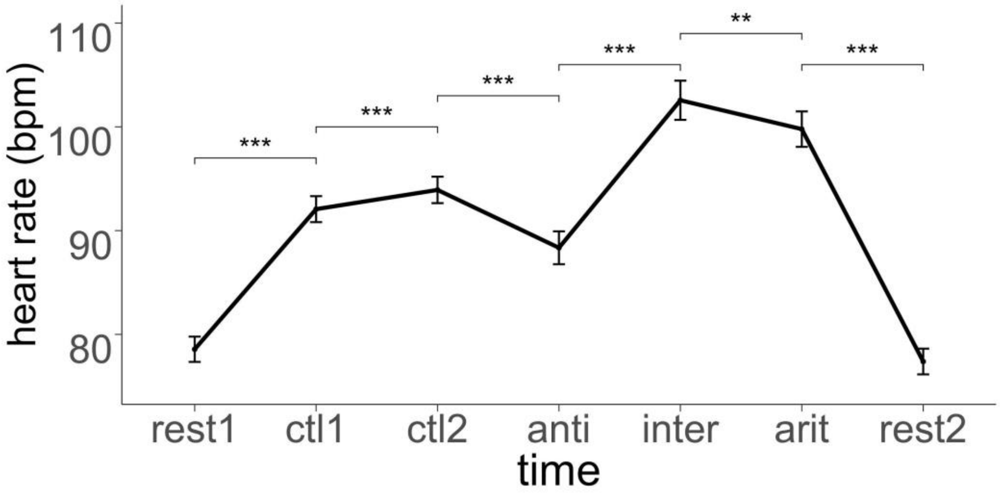

Figure S1. Heart rate in beats per minute (BPM) dependent on time (rest1 = resting-state measurement 1, ctl1 = control task 1, ctl2 = control task 2, anti = anticipation phase, inter = job interview, arit = arithmetic task, rest2 = resting-state measurement 2). Error bars indicate standard errors (*$p < .05$, **$p < .01$, ***$p < .001$).

Salivary cortisol

We investigated whether the stress induction was reflected by changes in salivary cortisol. Calculating mahalanobis distances, we identified four subjects´ cortisol values as multivariate outliers and therefore excluded them in our following analyses. Due to missing trait rumination and social anxiety values additionally two subjects were excluded from these analyses. From initially plotting the data, we suggested a quadratic relationship of subjective stress ratings and time and therefore tested a linear model against a quadratic model using a Likelihood-ratio-test. Results revealed a significantly better fit of the quadratic model, $\chi^2(1) = 122.9$, $p < .001$, which is the reason why in the following, we will be analyzing time as a linear and quadratic factor. Fitting the basic model, time yielded a significant predictor as linear as well as quadratic term (see table S1) and remained significant even in the more complex models. In model 2, we observed significant interactions of LSAS with both time factors. Analogous, the interaction terms with both time factors and RRS also were significant in model 3. In model 4, we observed the same pattern as before with both interactions of LSAS and time got significant but not the main effect of RSS. Comparably, in model 5 again both interaction terms were significant, but not the control variable LSAS (see table S1). Descriptively, we found salivary cortisol as a parameter of stress to be increased in case of higher social anxiety levels as well as in case of higher trait rumination. In the most complex model (model 6), including all possible interaction effects, beside the time variable only the interaction of time as quadratic term and the RRS yielded significance (see table S1). However, this last model needs to be interpreted with caution, due to effects of collinearity, as the two variables RRS and LSAS are moderately correlated ($r = .519$***) and therefore an interpretation of the slope coefficients as well as of the changes in one predictor, while holding the other constant, is not easily possible [116-118].

In order to investigate the effect of time, we used planned contrasts. They yielded significant increases in salivary cortisol from 30 min before the stress induction (TSST) to immediately (0 min) after the TSST ($t(85) = -5.043$, $p < .001$, $d = -0.544$) and from 0 min to 15 min after the TSST ($t(85) = -6.051$, $p < .001$, $d = -0.652$), as well as significant decreases from 15 min to 30 min post TSST ($t(85) = 6.697$, $p < .001$, $d = 0.722$), from 30 min to 45 min post TSST ($t(85) = 6.504$, $p < .001$, $d = 0.701$) and from 45 min to 60 min post TSST ($t(85) = 3.625$, $p < .01$, $d = 0.391$) (see figure S2).

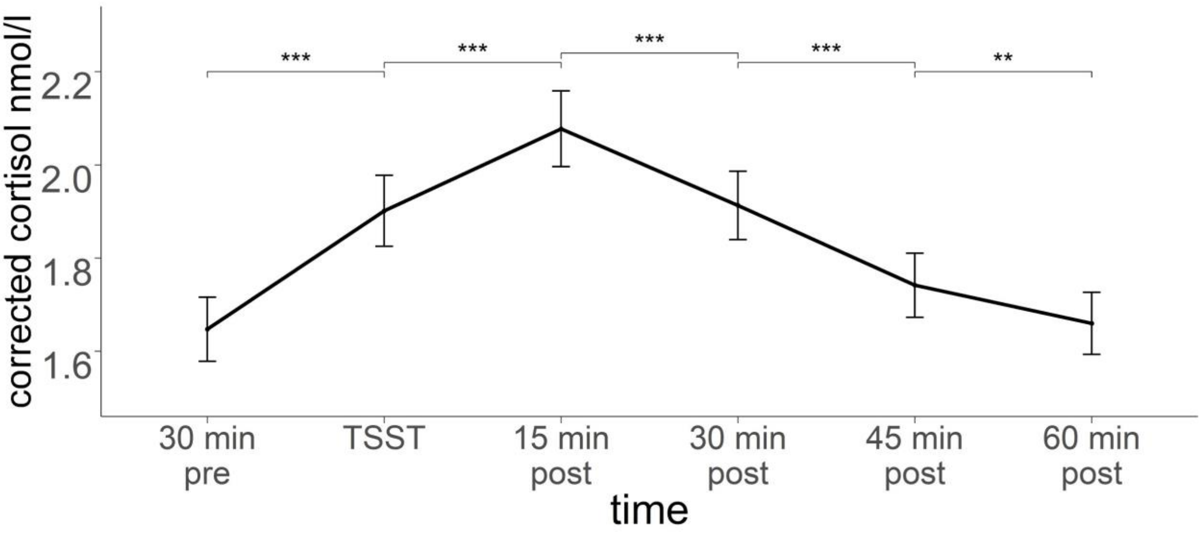

Figure S2. Corrected salivary cortisol in nmol/l dependent on time corresponding to minutes previous or after the stress induction. Time points on the x-axis refer to the stress induction via the TSST. Error bars indicate standard errors (*p < .05, **p < .01, ***p < .001).

Table S1

*Results of the Mixed Models exploring the association between corrected salivary cortisol, social anxiety (LSAS) and trait rumination (RRS) (DV = dependent variable; AIC = Akaike Information Criterion; BIC = Bayesian-Information-Criterion; R^2 = variance explained by the fixed effects). Significant results are shaded whereas darker shadows mean higher significance scores. #p < .1, *p < .05, **p < .01, ***p < .001*

Dependent variables	Salivary cortisol					
	Model 1: Basic model	Model 2: Basic model + LSAS	Model 3: Basic model + RRS	Model 4: Basic model + LSAS while correcting for RRS	Model 5: Basic model + RRS while correcting for LSAS	Model 6: Interaction model
Intercept	1.630*** (0.073)	1.628*** (0.072)	1.631*** (0.073)	1.628*** (0.072)	1.629*** (0.072)	1.664*** (0.812)
Time	0.480*** (0.05)	0.481*** (0.05)	0.479*** (0.05)	0.481*** (0.05)	0.479*** (0.05)	0.472*** (0.051)
$Time^2$	-0.179*** (0.025)	-0.179*** (0.025)	-0.129*** (0.025)	-0.179*** (0.025)	-0.179*** (0.025)	-0.177*** (0.025)
$Time*Time^2$	0.017*** (0.003)	0.017** (0.003)	0.018*** (0.003)	0.017*** (0.003)	0.017*** (0.003)	0.017*** (0.003)
LSAS		0.139# (0.071)		0.142# (0.083)	0.112 (0.08)	0.165# (0.095)
Time*LSAS		-0.056* (0.022)		-0.056* (0.022)		-0.036 (0.029)
$Time^2$*LSAS		0.012** (0.004)		0.012** (0.004)		0.008 0.006)
RRS			0.097 (0.074)	-0.006 (0.083)	0.036 (0.086)	0.014 (0.088)
Time*RRS			-0.071** (0.023)		-0.071** (0.034)	-0.053# (0.027)
$Time^2$*RRS			0.015*** (0.004)		0.015*** (0.004)	0.011* (0.005)
Time*LSAS*RRS						0.016 (0.024)
$Time^2$*LSAS*RRS						-0.004 (0.005)
AIC	327.7	322.9	321.2	324.9	321.3	328.3
BIC	353.0	360.9	359.3	367.1	363.6	391.6
R^2	.045	.072	.054	.073	.073	.081

Table S2

Items state rumination included adapted items from the RRS [9], ARSQ [100], PTQ [101,102]. Subjects were instructed to rate if the items were in line with their mental state during the last 10 minutes

German	English
Konnte ich meine Gedanken nur mühsam festhalten.	I had difficulties holding on to my thoughts.
Ich dachte darüber nach, warum ich mich in bestimmten Situationen falsch verhalten habe.	I thought about why I acted wrong in certain situations.
Ich fragte mich, warum ich Probleme habe, die andere nicht haben.	I thought why I have problems other people don´t have.
Ich fragte mich, womit ich meine momentane Lebenssituation verdient habe.	I thought about whereby I deserved my current life situation.
Ich dachte darüber nach, warum ich die Dinge nicht besser in den Griff bekomme.	I thought why I can´t handle things better.
Ich dachte an all meine Defizite und Misserfolge, Macken und Fehler.	I thought about all my shortcomings, failings, faults, mistakes.
Ich dachte an vergangene Situationen die ich bereue.	I thought about past situations that I regret.
Ich war von meinen Sorgen und Problemen stark vereinnahmt.	I was consumed by my problems and worries.

Table S3

Regions of interest and corresponding probesets and channels, extrapolated based on the Colin 27 template [120]. For probeset placement see also Figure S3

region of interest	probeset	corresponding channels
left inferior frontal gyrus (lIFG)	left frontal	6 7 9
left dorsolateral prefrontal cortex (lDLPFC)	left frontal	10 11 12
right inferior frontal gyrus (rIFG)	right frontal	18 19 21
right dorsolateral prefrontal cortex (rDLPFC)	right frontal	20 23 24

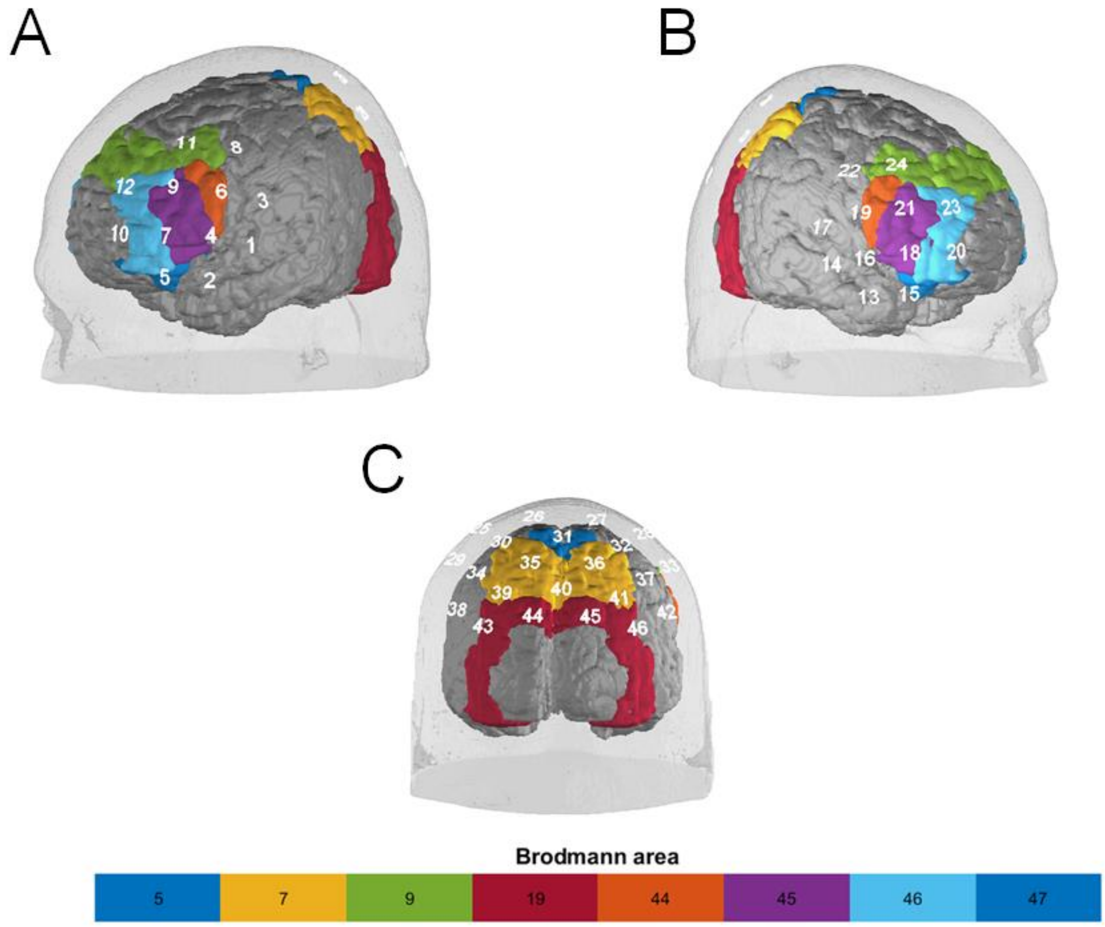

Figure S3. Used fNIRS probeset placement and related Brodmann areas (A = left frontal, B = right frontal, C = parietal). For probeset placement see also table 2.
